# Hemoperitoneum due to Splenic Laceration Caused by Colonoscopy: A Rare and Catastrophic Complication

**DOI:** 10.1155/2014/985648

**Published:** 2014-03-17

**Authors:** Shiao-Han Chen, Jiann-Ruey Ong, Hon-Ping Ma, Po-Shen Chen

**Affiliations:** Department of Emergency, Shuang-Ho Hospital, Taipei Medical University, No. 291, Zhongzheng Road, Zhonghe District, New Taipei City 235, Taiwan

## Abstract

Numerous studies suggest that in asymptomatic patients, routine follow-up CT is not indicated due to the insignificant findings found on these patients. A 53-year-old man, who denied any underlying disease before, underwent colonoscopy for routine health examination. Sudden onset of abdominal pain around left upper quarter was mentioned at our emergency department. Grade II spleen laceration was found on CT scan. Splenic injury was found few hours later on the day of colonoscopy. It might result from the extra tension between the spleen and splenic flexure which varies from different positions of patients.

## 1. Background

Colonoscopy is a relatively safe procedure for the diagnosis and treatment. The major complications are bleeding, which is 4.8 per 1000 colonoscopies mostly after biopsy and polypectomy, and perforation, which is 0.9 per 1000 colonoscopies, mostly due to excessive looping and overdistension of cecum [1]. Splenic injury is a rare and catastrophic consequence after colonoscopy. Approximately 66 cases have been reported in the literature to date [2]. The mechanism of splenic injury followed by colonoscopy is believed due to the stretching splenocolic ligament that resulted from the movement of colonoscope or the formation of loop at the splenic flexure [3]. Developed adhesions between splenic flexure of colon and spleen would increase the immobility of spleen. Such adhesion could result from prior abdominal surgery, inflammatory bowel disease, and pancreatitis. Additional traction on splenocolic ligament could be exerted by the external compression on the left side of abdomen,in order to prevent the loop formation of colonoscope. Such movement on a patient with adhesion between spleen and splenic flexure would increase the risk of splenic injury. Other risk factors include difficult insertion, looping inside left colon, and splenomegaly [3]. However, splenic injury could cause mortality if not diagnosed earlier. In the clinical presentation of splenic injury, abdominal pain is the most common sign, which could be mistaken for common abdominal discomfort due to distension of colon. Referred pain to left shoulder, which is called Kehr's sign, could be presented. It is highly sensitive but not specific that it also can be presented in a patient after an uncomplicated colonoscopy [4]. A CT grading system for splenic injury was developed by the American Association for the Surgery of Trauma (AAST) to classify severity of splenic injury [1]. Controversy exists regarding appropriate nonoperative management of splenic injury depending on imaging strategies [5]. Numerous studies suggest that in asymptomatic patients routine follow-up CT is not indicated, due to insignificant findings found on these patients [6–9].

## 2. Case Presentation

A 53-year-old man, who denied any underlying disease before, underwent colonoscopy for routine health examination. Several hours later, sudden onset of abdominal pain around left upper quarter was mentioned. He came to our emergency department with chief complaints of abdominal pain and left shoulder pain. On arrival, his vital signs were as following: body temperature 36°C, pulse rate 82 beats per minute, respiratory rate 22 breaths per minute, and blood pressure 110/76 mmHg. The physical examination revealed pink conjunctiva with unremarkable cardiovascular and pulmonary findings. On palpation, tenderness over the epigastric and left upper quadrants of his abdomen was noted. Involuntary muscle guarding was also noted.

**Figure 1:**
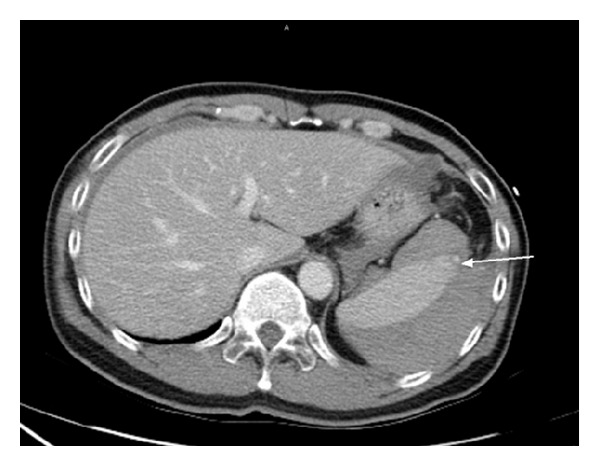
Massive hyperdense ascites surrounded around the spleen without free air.

**Figure 2:**
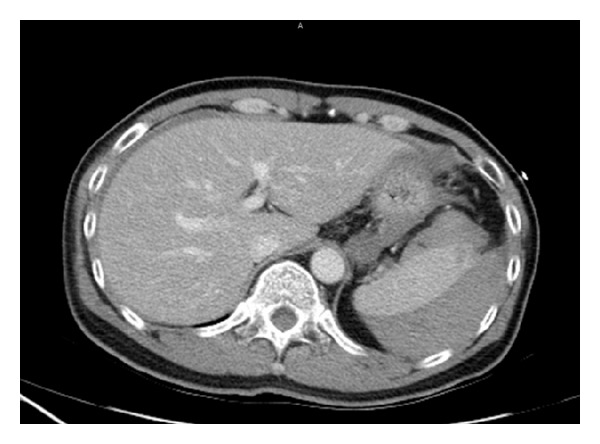
Massive hyperdense ascites surrounded around the spleen without free air.

Laboratory examination showed 17000/uL white blood cell (WBC) and normal hemoglobin (14.3 gm/dL). His ECG showed sinus rhythm with RBBB and his chest radiograph was normal without evidence of free air. During the observation period at ED, suddenly his systolic blood pressure was dropped to 90 mmHg with sinus tachycardia. Ongoing severe abdominal pain was mentioned. He received abdominal computed tomography (CT) under the initial impression of hollow organ perforation with septic shock. However, CT scan showed massive hyperdense ascites surrounded around the spleen without free air (Figures 1 and 2). Grade II of spleen laceration was found on this CT scan. Therefore, spleen laceration with hemoperitoneum as the complication of colonoscopy was impressed. General Surgeon was consulted at the same time. Due to the fact that severity of spleen laceration is grade II, conservative treatment was suggested. He received blood transfusion with PRBC 2U; then, he was admitted to ward. After admission, his hemodynamic status was stable with improving symptoms. So, he was discharged from our hospital 3 days later. However, LUQ pain was noted on the same day of discharge, so he came back to our ED immediately. Abdominal and pelvic CT was arranged again with progressing ascites compared to previous study. Grade III of spleen laceration was noted. General surgeon was consulted and splenectomy was indicated. He received splenectomy after admission. According to the procedural record of his colonoscopy, it was performed smoothly without any significant technical difficulty. Splenic injury was found few hours later on the day of colonoscopy. It might result from the extra tension between the spleen and splenic flexure which varies from different positions of patients.

The patient was discharged under the stable vital signs with improving symptoms. However, the follow-up abdominal CT scan showed progressed hemorrhage. In the end, splenectomy was arranged in order to prevent further worsening outcome. Whether routine follow-up CT scan is indicated even in stable asymptomatic patient may warrant further studies to have conclusion.

## 3. Conclusion

In our case, the patient without any systemic disease before had splenic laceration after the routine screening colonoscopy. We report this case in order to remind physicians that even healthy people could have this catastrophic complication after colonoscopy. Most of complications after colonoscopy were bleeding and perforation. Spleen injury was rare. Most clinical presentation was asymptomatic or may be a complaint of abdominal pain. Then, if hemodynamic change occurs, it may be a splenic injury. CT scan is the modality of choice to diagnose splenic injury and the most sensitive methods to diagnose this rare condition.
